# Ischemic stroke mortality and time for hospital arrival: analysis of the first 90 days

**DOI:** 10.1590/1980-220X-REEUSP-2022-0309en

**Published:** 2023-04-14

**Authors:** Mariana de Almeida Moraes, Pedro Antônio Pereira de Jesus, Ludimila Santos Muniz, Greice Alves Costa, Larissa Vitória Pereira, Letícia Melquiades Nascimento, Carlos Antônio de Souza Teles, Camila Antunes Baccin, Fernanda Carneiro Mussi

**Affiliations:** 1 Universidade Federal da Bahia, Escola de Enfermagem, Salvador, BA, Brazil.; 2 Universidade Federal da Bahia, Instituto de Ciências da Saúde, Salvador, BA, Brazil.; 3 Universidade Estadual de Santa Cruz, Ilhéus, BA, Brazil.; 4 Fiocruz, Instituto Gonçalo Muniz, Salvador, BA, Brazil.; 5 Universidade Estadual de Feira de Santana, Feira de Santana, BA, Brazil.; 6 Universidade Federal de Santa Catarina, Florianópolis, SC, Brazil.

**Keywords:** Stroke, Mortality, Nursing, Cohort Studies, Accidente Cerebrovascular, Mortalidad, Enfermería, Estudios de Cohortes, Acidente Vascular Cerebral, Mortalidade, Enfermagem, Estudos de coortes

## Abstract

**Objective:**

To analyze the association between time of arrival at a reference hospital and mortality of people with ischemic stroke.

**Method:**

Descriptive and inferential statistics were used. Modifying and confounding variables between time of arrival and mortality were observed in the multivariate analysis. The Akaike Information Criterion was used to choose the model. Statistical significance of 5% and risk correction using the Poisson Model were adopted.

**Results:**

Most participants arrived within 4.5 hours of symptom onset or wake up stroke to the referral hospital and 19.4% died. The score of the *National Institute of Health Stroke Scale* was a modifier. In the multivariate model stratified by scale score ≥14, arrival time >4.5h was associated with lower mortality; and age ≥60 years and having Atrial Fibrillation, to higher mortality. In the model stratified by score ≤13, previous Rankin ≥3, and presence of atrial fibrillation were predictors of mortality.

**Conclusion:**

The relationship between time of arrival and mortality up to 90 days was modified by the *National Institute of Health Stroke Scale*. Prior Rankin ≥3, atrial fibrillation, time to arrival ≤4.5h, and age ≥60 years contributed to higher mortality.

## RESUMO

**Objetivo:**

Analisar a associação entre tempo de chegada a hospital de referência e mortalidade de pessoas com Acidente Vascular Cerebral isquêmico.

**Método:**

Foram utilizadas estatísticas descritivas e inferenciais. Variáveis modificadoras e confundidoras entre tempo de chegada e mortalidade foram verificadas na análise multivariada. O Critério de Informação de Akaike foi utilizado para escolha do modelo. Adotou-se significância estatística de 5% e correção de risco pelo Modelo de Poisson.

**Resultados:**

A maioria dos participantes chegou em até 4,5 horas do início dos sintomas ou *wake up stroke* ao hospital de referência e 19,4% morreram. A pontuação da *National Institute of Health Stroke Scale* foi um modificador. No modelo multivariado estratificado por pontuação da escala ≥14, o tempo de chegada >4,5h estava associado a menor mortalidade; e idade ≥60 anos e ter Fibrilação Atrial, a maior mortalidade. No modelo estratificado por pontuação ≤13, Rankin prévio ≥3 e ter fibrilação atrial foram preditores de mortalidade.

**Conclusão:**

A relação entre tempo de chegada e mortalidade até 90 dias foi modificada pela pontuação da *National Institute of Health Stroke Scale*. Rankin prévio ≥3, fibrilação atrial, tempo de chegada ≤4,5h e idade ≥60 anos contribuíram para maior mortalidade.

## RESUMEN

**Objetivo:**

Analizar la asociación entre el tiempo de llegada a un hospital de referencia y la mortalidad de las personas con accidente cerebrovascular isquémico.

**Método:**

Fueron usadas estadísticas descriptivas e inferenciales. En el análisis multivariado se verificaron variables modificadoras y confusoras entre el tiempo de llegada y la mortalidad. Para la elección del modelo se utilizó el Criterio de Información de Akaike. Se adoptó la significación estadística del 5% y la corrección del riesgo mediante el Modelo de Poisson.

**Resultados:**

La mayoría de los participantes llegaron dentro de las 4,5 horas posteriores al inicio de los síntomas o *wake up stroke* al hospital de referencia y el 19,4% fallecieron. La puntuación de la e*scala National Institute of Health Stroke Scale* fue un modificador. En el modelo multivariante estratificado por puntaje de escala ≥14, el tiempo de llegada >4,5 h se asoció con una menor mortalidad; y edad ≥60 años y con Fibrilación Auricular a mayor mortalidad. En el modelo estratificado por puntaje ≤13, Rankin previo ≥3 y tener fibrilación auricular fueron predictores de mortalidad.

**Conclusión:**

La relación entre tiempo de llegada y mortalidad hasta los 90 días fue modificada por puntaje de la escala *National Institute of Health Stroke Scale*. Rankin previo ≥3, fibrilación auricular, tiempo hasta la llegada ≤4,5 h y la edad ≥60 años contribuyeron a una mayor mortalidad.

## INTRODUCTION

Stroke is currently the second leading cause of death in the world^(1)^. It is estimated that by 2060 the event will continue in this position and be responsible for 10.6% of predicted deaths for the year and 12.8% of deaths in Brazil^(2)^.

Variations in the coefficient of mortality from stroke have been related to socioeconomic conditions^(3–5)^ and regional differences^(6,7)^. A study of the *Global Burden of Disease* showed that the reduction in cerebrovascular mortality was uneven within the states, being always more incisive in the federative units with better socioeconomic indicators^(3)^. This reveals a direct relationship between cerebrovascular disease and the worst indicators of social and economic development.

A systematic review that synthesized 12 population-based studies from 10 low- and middle-income countries and 44 studies from 18 high-income countries found significant disparities in stroke incidence trends among these countries. Over the last four decades, the incidence of stroke has decreased by 42% in high-income countries, but has increased by more than 100% in those with low incomes^(8)^. However, the absolute number of people annually affected, who live after the event and die is increasing worldwide^(4,8)^.

In 2017, in Latin American countries, there were 5.5 million people who survived strokes, 0.6 million new strokes, more than 0.26 million deaths, and approximately 5.5 million of years of life lost due to disability by stroke^(9)^. In Brazil, in 2020, 34,369 people died from this event^(10)^.

Mortality in the first 30 days after ischemic stroke is about 10%, reaching 40% at the end of the first year^(4)^, and the survival depends on early treatment^(11)^. The time elapsed between the onset of symptoms and the search for care at an appropriate health service is a determining factor in the clinical course of the stroke, since the effectiveness of reperfusion therapies is time-dependent^(12)^.

Despite advances in the treatment of the acute phase of stroke, mortality rates remain high^(5)^. These data suggest that there are substantial problems in establishing effective therapies and care, in primary and secondary event prevention strategies, or a combination of these factors^(9)^.

In addition to the problems related to the delay in the search for an adequate health service, which implies in delay or impossibility of the institution of thrombolytic therapy^(4)^, studies in populations with stroke also reported that low levels of income and education were related to unfavorable prognosis^(7,13)^. In addition, a recent international literature review highlighted racial and ethnic disparities in people with stroke. Reasons for these disparities included a higher incidence of inherited and acquired risk factors, gaps in knowledge about recognizing warning signs, delay in presenting for timely treatment, and less access to care. Also according to the authors, black individuals presented arterial hypertension at a younger age and with greater severity compared to white people^(14)^. A study on cardiovascular statistics in Brazil highlighted that stroke is more frequent, more severe, and has greater mortality in black people^(4)^. However, there is a lack of national studies analyzing racial differences related to mortality.

Other variables associated with differences in mortality from the disease included sex, age^(15)^, stroke severity assessed by the *National Institute of Health Stroke Scale* (NIHSS)^(5)^, length of stay in the hospital^(7)^, and hospitalization in a specialized unit (*UAVC*)^(16)^.

Despite advances in therapies and epidemiological knowledge of stroke, no cohort studies were found that evaluated the association between time of arrival at a reference hospital and mortality up to 90 days due to stroke in Brazil as the main independent variable. Therefore, knowing variables that influence the association between the time of arrival at a reference hospital and mortality in stroke is considered important. Obtaining this knowledge will contribute to clinical practice as it will guide education, management and health care strategies to reduce the presentation time of people affected by stroke, increasing the chances of reversing the clinical picture and of better outcomes^(17)^.

Therefore, the objective of this study was to analyze the association between time for arrival at a reference hospital in neurology (TARH) and mortality of people with ischemic stroke.

## METHODS

### Design of Study and Local

This is a cohort, prospective study carried out in a public hospital in the state of Bahia, Brazil, level III certification for stroke patients.

### Sample

The access population consisted of 320 people diagnosed with acute ischemic stroke, aged at least 18 years, hospitalized at the investigation site from March to October 2019. Of these, 12 were excluded for presenting symptoms that prevented verbal communication and for not having companions to answer the research questions, or being more than 10 days away from the attack, due to the possibility of recall bias. Nine participants were lost to follow-up because they were not contacted by phone calls. Therefore, the sample of 299 participants in the cohort was reached.

### Data Collection Instruments

#### Instrument I – Sociodemographic and Clinical Characterization Data

For data collection, an instrument with multiple-choice and semi-structured questions for sociodemographic (age in years, sex, self-declared race/color, education, monthly family income in minimum wages, and marital status) and clinical (Previous Rankin, NIHSS score, presence of previous atrial fibrillation (AF), diabetes mellitus (DM), systemic arterial hypertension (SAH), dyslipidemia, stroke and/or acute myocardial infarction (AMI), smoking habits, performance of thrombolysis, and hospitalization in possible units) characterization.

The instrument also included space for recording the date and time of onset of symptoms or Wake Up Stroke, and date and time of arrival at the study site, which allowed the calculation of the independent variable TARH (time elapsed between the onset of symptoms or Wake Up Stroke and arrival at the study site). It also contained space for hospital discharge records, including cases of death.

#### Instrument II – Rankin Scale

Another instrument used was the Rankin Scale, translated and culturally adapted version for Brazil and validated for application via telephone^(18)^. This scale was applied to check the presence of functional capacity prior to the current event (Premorbid Rankin).

#### Instrument III – Telephone Call Protocol

The protocol was designed to standardize the telephone approach by the researchers. The instrument also had items to record the participants’ identification and the occurrence of death within 90 days of the attack, with identification of its date and place.

### Data Collection Procedures

Data collection took place in three phases. Phase I took place from March to October 2019, Phase II from March 2019 to January 2020, and Phase III from June 2019 to January 2020.

Phase I corresponded to the identification of eligible participants in the inpatient units of the study site, explanation of the objectives and importance of the study, and invitation to participate. Following acceptance, the participants signed the Free and Informed Consent Form. In this phase, sociodemographic and clinical data (pre-morbid Rankin, previous stroke and acute myocardial infarction, smoking, first health service sought) were collected through an interview, except for admission NIHSS, date and time of arrival at the study site, the presence of AF, DM, SAH, dyslipidemia, time of arrival at the study site, and performance of thrombolysis, which were obtained from the medical records. In situations where the participant did not have the clinical, cognitive, and/or emotional conditions to interact with the researcher, the approach was made to his/her companion.

Phase II corresponded to the follow-up of the participants during hospitalization, aiming to identify the hospitalization units sought in the study site, and the possible occurrence of death. For this, the researchers monitored the records of the nursing team shift changes, and the admission and discharge censuses of all units of the study. In this phase, clinical data not identified in phase I were also collected from the medical record. All information obtained was described in Instrument I – Sociodemographic and clinical characterization data.

Phase III corresponded to the follow-up of the participants up to 90 days after the attack. For those who were discharged from the hospital, contact was made via telephone after completion of three months of the event. For this phase, Instrument III – Telephone call protocol was filled out.

### Data Treatment and Analysis

Clinical, sociodemographic, and TARH categorical variables were analyzed in absolute and relative frequencies. TARH, the main independent variable, was dichotomized into ≤ 4.5 hours and > 4.5 hours from onset of symptoms or Wake up Stroke. The therapeutic window for performing thrombolysis was used as a reference for this categorization^(12)^. The dependent variable was death.

Then, a bivariate analysis was performed to verify the association of sociodemographic, clinical, and TARH variables with death using Pearson’s chi-square test or Fisher’s exact test. Variables whose associations showed p-value ≤ 0.20 were tested as potential interaction variables (modifiers) between TARH and death. The interaction term that was significant (p-value ≤ 0.20) was the NIHSS score, the variable being taken to the multivariate analysis with the variables that were statistically associated with death in the bivariate analysis. In this analysis, it was observed that this interaction term maintained statistical significance, indicating the stratification of the model.

The multivariate logistic regression analysis was carried out, with all variables of the complete model, except for the modifier (NIHSS), and the beta-adjusted measure being obtained. Therefore, the potential confounders of the association between the main and dependent independent variable were observed, by comparing the reduced models, which tested each specific variable, obtaining the respective association measures (beta). A confounding variable was considered to be the one whose removal generated a difference between the beta of the full model and the reduced model greater than or equal to 5.0%.

Next, the identified confounding variables (age, sex, admission to the stroke specialized unit, AF, and previous Rankin) composed the complete robust multivariate logistic regression model together with the independent variable. To choose the best model, the Akaike Information Criterion (AIC) and statistical significance of 5% were used. After defining the best model, the chosen variables composed models in each specific stratum of the NIHSS variable score (≤ 13 and ≥ 14), the identified modifier variable.

Considering that the outcome mortality was common in the group studied, risk correction was adopted by applying the (robust) Poisson Model.

Analyses were performed using STATA version 22.0.

### Ethical Aspects

A study that is part of the Matrix Project “Factors associated with disability and mortality due to Ischemic Stroke and times of access to treatment”, approved by the Ethics Committee, Opinion No. 3.159.694. The study complies with Resolutions 466/12 and 580/18 of the National Health Council.

## RESULTS

Of the sample consisting of 299 people, the predominance was of women (50.8%), age group ≥ 60 years (69.2%), self-declared black race/color (84.2%), no partner (53.4%), with up to eight years of study (67.8%), and monthly family income of up to three minimum wages (89.7%).

As for the TARH, 58.9% arrived within 4.5 hours of the onset of symptoms or *Wake Up Stroke.*

Death was found in 19.4% of the sample, with 38 being in-hospital deaths and 20 after hospital discharge.

In the bivariate analysis, a statistically significant difference at 5% was observed between death and age group, sex and self-declared race/color. Participants aged 60 or over, female, and non-black died in a higher proportion (Table 1).

**Table 1.** Association of sociodemographic variables and participant mortality – Salvador, Bahia, Brazil, 2021.

Sociodemographic variables	Sample n (%)	Death		p value
		Yes n (%)	No n (%)	
**Age group** n = 299				
<60 years	92 (30.8)	10 (10.9)	82 (89.1)	**0.010**
≥60 years	207 (69.2)	49 (23.7)	158 (76.3)	
**Sex** n = 299				
Male	147 (49.2)	22 (15.0)	125 (85.0)	**0.042**
Female	152 (50.8)	37 (24.3)	115 (75.7)	
**Self-declared race/color** n = 298*				
Non black	47 (15.8)	13 (27.7)	34 (72.3)	**0.141**
Black	251 (84.2)	46 (18.3)	205 (81.7)	
**Marital status** n = 298*				
With a partner	139 (46.6)	26 (18.7)	113 (81.3)	0.760
Without a partner	159 (53.4)	32 (20.1)	127 (79.9)	
**Level of Education** n = 295***				
More than 8 years of study	95 (32.2)	16 (16.8)	79 (83.2)	0.401
Up to 8 years of study	200 (67.8)	42 (21.0)	158 (79.0)	
Family monthly income** n = 292***				
≤3 minimum wages	30 (10.3)	51 (19.5)	211 (80.5)	0.712
>3 minimum wages	262 (89.7)	5 (16.7)	25 (83.3)	

*p value of dichotomous variables obtained by the t-test. Remaining values obtained by Anova. **Minimum wage for 2020: BRL 1040.00. ***Reduced sample for this variable due to data not reported during the interview with the companion.

Regarding the participants’ clinical characteristics (Table 2), the most prevalent comorbidity was systemic arterial hypertension (78.3%), followed by dyslipidemia (31.5%), diabetes mellitus (28.9%), and atrial fibrillation (10.6%). As for previous events, 33.2% reported stroke and 11.4% myocardial infarction. Regarding smoking, 40.1% reported being smokers or former smokers. Regarding previous functional incapacity, 91.6% of the sample had a previous Rankin between 0 and 2.

**Table 2.** Association between TARH and participants’ clinical characteristics and mortality – Salvador, Bahia, Brazil, 2021.

Clinical variables	Sample n (%)	Death (sample = 299)		
		Yes (%)	No (%)	p value*
**Time of arrival at the site**				
≤4.5 hours	176 (58.9)	42 (23.9)	134 (76.1)	**0.032**
>4.5 hours	123 (41.1)	17 (13.8)	106 (86.2)	
**Systemic Arterial Hypertension**				
Yes	234 (78.3)	47 (20.1)	187 (79.9)	0.771
No	65 (21.7)	12 (18.5)	53 (81.5)	
**Dyslipidemia** n = 298***				
Yes	94 (31.5)	14 (14.9)	80 (85.11)	**0.176**
No	204 (68.5)	44 (21.6)	160 (78.4)	
**Diabetes mellitus** n = 294***				
Yes	85 (28.9)	19 (22.4)	66 (77.6)	0.471
No	209 (71.1)	39 (18.7)	170 (81.3)	
**Atrial fibrillation** n = 284***				
Yes	30 (10.6)	13 (43.3)	17 (56.7)	**0.000**
No	254 (89.4)	40 (15.8)	214 (82.2)	
**Previous stroke** n = 298***				
Yes	99 (33.2)	26 (26.3)	73 (73.7)	**0.048**
No	199 (66.8)	33 (16.6)	166 (83.4)	
**Previous AMI** n = 297***				
Yes	34 (11.4)	7 (20.6)	27 (79.4)	0.911
No	263 (88.6)	52 (19.8)	211 (80.2)	
**Smoking**				
Never smoked	179 (59.9)	35 (19.6)	144 (80.4)	0.729
Smoker	38 (12.7)	6 (15.8)	32 (84.2)	
Former smoker	82 (27.4)	18 (22.0)	64 (78.0)	
**Prior Rankin** n = 296***				
0–2 (Asymptomatic to mild deficiency)	271 (91.6)	44 (16.2)	227 (83.8)	**<0.001**
3–5 (Moderate to severe disability)	25 (8.4)	12 (48.0)	13 (52.0)	
NIHSS** n = 248***				
≤5	63 (25.4)	1 (1.6)	62 (98.4)	**<0.001**
6 to 13	109 (44.0)	11 (10.1)	98 (89.9)	
≥14	76 (30.6)	30 (39.5)	46 (60.5)	
**Venous thrombolysis**				
Yes	78 (26.1)	15 (19.2)	63 (80.8)	0.897
No	221 (73.9)	44 (19.9)	177 (80.1)	
**Admission at UAVC**				
Yes	213 (71.2)	33 (15.5)	180 (84.5)	**0.023**
No	86 (28.8)	26 (30.2)	60 (69.8)	
**First health service sought**				
Site of study	46 (15.4)	7 (15.2)	39 (84.8)	0.312
Mobile Emergency Care Service (*SAMU)*	70 (23.4)	18 (25.7)	52 (74.3)	
Other service	183 (61.2)	34 (18.6)	149 (81.4)	

*p value of the dichotomous variables obtained by Pearson’s chi-square, except for the variable NIHSS, first health service sought and smoking, whose test was Fisher’s Exact. **NIHSS = National Institute of Health Stroke Scale. ***Reduced sample for this variable due to lack of knowledge about the clinical condition by the participant and lack of information in the medical record.

Most participants first sought another health service other than the reference hospital (84.6%), did not undergo venous thrombolysis (73.9%), was at the UAVC at some point during hospitalization (71.2%), and scored higher or equal to six in the NHISS at admission (74.6%).

The bivariate analysis (Table 2) showed a statistically significant association at 5% between TARH and death within 90 days of the attack, with a higher percentage of deaths being observed for those who arrived at the reference hospital before 4.5 hours after the onset of symptoms or *wake up stroke*. There was also a higher percentage of death for participants with atrial fibrillation, history of previous stroke, pre-morbid Rankin score between 3 and 5, NIHSS ≥ 14 and not admitted to the UAVC, with these associations being statistically significant (Table 2).

Variables with a statistically significant difference of up to 20% in the bivariate analyses (age, sex, self-reported race/color, atrial fibrillation, previous stroke, previous Rankin, NIHSS score and admission at UAVC) were tested as potential modifiers between TARH and mortality, with the NIHSS score being identified.

Then, all variables with a statistically significant difference of 20% in the bivariate analyses (Tables1 and 2), with the exception of the NIHSS score, were tested as potential confounders of the association of main interest, with the identification of age, sex, admission at UAVC, AF, and previous Rankin. Thus, these variables made up the complete model and the models stratified by the NIHSS score of ≤13 or ≥14 (Table 3).

**Table 3.** Association of TARH and confounding variables with death shown in the full regression model and in the robust regression models stratified by NIHSS – Salvador, Bahia, Brazil, 2021.

Variables	Model 1 (n = 237) OR (CI 95%)	Model 2 (n = 166) OR (CI 95%)	Model 3 (n = 71) OR (CI 95%)
AT > 4.5h	0.48 (0.22 – 1.08)	1.46 (0.41 – 5.22)	0.33 (0.11 – 0.98)
Age ≥ 60 years	2.04 (0.91 – 4.60)	1.60 (0.34 – 7.47)	2.75 (1.09 – 6.94)
Female Sex	1.72 (0.91 – 3.25)	2.87 (0.77 – 10.76)	1.18 (0.64 – 2.16)
Not having been admitted at the UAVC	1.46 (0.83 – 2.55)	0.54 (0.10 – 3.02)	1.18 (0.68 – 2.05)
To have atrial fibrillation	2.08 (1.19 – 3.62)	3.28 (1.05 – 10.31)	1.64 (1.01 – 2.68)
Prior Rankin 3 to 5	2.32 (1.32 – 4.07)	5.71 (2.14 – 15.26)	1.21 (0.67 – 2.18)
AIC	183.6114	83.27629	86.77243

Model 1 = Complete (Gross); Model 2 = stratified by NIHSS ≤ 13; Model 3 = stratified by NIHSS ≥ 14.

In the complete model (n = 237), with all variables analyzed simultaneously, no statistical significance was identified for the TARH (OR 0.48, CI 95%: 0.22 – 1.08). However, people with AF died 2.1 times more than those without this comorbidity (OR 2.08, CI 95%: 1.19 – 3.62) and those with a previous Rankin from 3 to 5 died 2.3 times more than those with a previous Rankin from 0 to 2 (OR: 2.32; CI 95%: 1.32 – 4.07).

A similar result was found in Model 2 (n = 166), stratified by NIHSS ≤ 13, in which no statistical significance was identified for the TARH (RP 1.46, CI 95%: 0.41 – 5.22), with all variables analyzed simultaneously. However, people with AF died 2.1 times more than those without this comorbidity (OR 2.08, CI 95%: 1.19 – 3.62) and those with a previous Rankin from 3 to 5 died 2.3 times more than those with a previous Rankin from 0 to 2 (OR: 2.32; CI 95%: 1.32 – 4.07).

In the model stratified by NIHSS ≥ 14 (n = 71), with all variables analyzed simultaneously, participants who arrived 4.5 hours after the onset of symptoms died less than those who arrived after this period, with this relationship being statistically significant (OR: 0.03; 95% CI: 0.11 – 0.98). Furthermore, participants aged 60 and over died almost three times as often as those under 60 (OR: 2.75; 95% CI: 1.09 – 6.94), and those with AF died 1.6 times more often than those without AF (OR: 1.64; 95% CI: 1.01 – 2.68).

## DISCUSSION

In this study, there was a slight predominance of women. Most participants were elderly, black, without a partner, and with low income and education, showing a sociodemographic profile similar to that found in another study conducted in the Northeast^(13)^.

As for the time of arrival at the reference hospital in neurology, more than half of the sample arrived within 4.5 hours of the onset of symptoms or *wake up stroke*, but still relevant percentage arrived out of the window. Arriving within the therapeutic window at a reference hospital in neurology is directly linked to the possibility of undergoing intravenous thrombolysis, which has time-dependent benefits. Early therapeutic intervention is extremely important to reverse ischemia and reduce the impact of morbidity and mortality in the face of stroke^(17,19)^. It is also important to highlight that appropriate therapeutic management instituted early, including etiological investigation, rehabilitation and clinical stability^(20)^, prescription of drugs according to the etiology of the ischemic stroke^(21)^, and admission to stroke units^(22)^ are related to better outcomes.

In this study, high mortality was observed, in about 20% of the sample, especially in the in-hospital environment. In a hospital study conducted in the Northeast and Southeast regions of Brazil (n = 962), mortality rates from ischemic stroke were lower, 7.0% after 10 days (95% CI, 5.3 – 8.8) and of 11.1% after 28 days^(23)^ (95% CI, 8.9 – 13.3). However, the period of death registration was shorter than the follow-up period in our study. International studies with people who suffered a stroke also observed mortality rates up to 90 days lower than this study (11.5 and 8.9%)^(12)^. However, another Brazilian study with 2407 patients also showed high (20.9%) in-hospital mortality due to stroke^(24)^. Analysis of the reduction in ischemic stroke mortality rates in Brazilian regions shows that they are always lower in the North and Northeast^(4)^.

The NIHSS score was considered a potential modifier of the association of interest, justifying the stratification of the models. This finding corroborates a Spanish study that revealed worse NIHSS scores as the main predictor of in-hospital mortality after stroke^(5)^ and an investigation carried out in Bahia that identified NIHSS as an independent predictor of death in 90 days^(25)^.

It should be noted that being a woman, although significant for higher mortality in the bivariate analysis, lost significance in the models when adjusted for all variables simultaneously. Dantas et al.^(15)^ evaluated hospitalizations in public services due to stroke in Brazil, from 2009 to 2016, and identified that men showed a significant association with participants’ survival. Other investigations also highlighted a worse outcome for women^(5,26)^. According to these studies, women were less likely to be admitted to a stroke unit than men, which could influence their outcomes^(5)^; and they more commonly reported non-stroke symptoms, thus being at risk of late recognition and delay in treatment^(26)^. An ecological study carried out in the southwest of Bahia showed that the number of deaths in the female population was higher than in the male population from 2013 to 2016, showing a continuous increase^(6)^.

Regarding the main independent variable, it was observed that the ratio of TARH ≥ 4.5 hours from the onset of symptoms or *Wake Up Stroke* with the outcome of death changed in the stratified models, as it was a predictor of higher mortality for those with NIHSS ≤ 13, but without statistical significance, and of lower mortality for those with NIHSS ≥ 14, with statistical significance, in the presence of the other variables. These results suggest that the reduction of pre-hospital delay influences the possibility of providing adequate therapy and the outcome after the event in people with mild to moderate stroke, highlighting the importance of immediate and qualified care for people with acute events, through well-defined flows and detailed procedures to ensure survival^(27)^. However, in more severe cases of stroke, early arrival at the emergency department was not associated with the survival of affected individuals.

In the most severe cases, the main predictors of mortality were age over 60 years and having atrial fibrillation. Older age was also highlighted as an important risk factor for stroke mortality in other studies^(23,24)^, with an increase in mortality proportional to age increase^(28)^. Reinforcing these findings, Dantas et al.^(15)^ also identified that younger age showed a significant association with participant survival.

The finding of AF as a predictor of higher mortality has been reinforced in other studies, such as the Brazilian cohort that showed a high burden of comorbidities as an independent predictor of prognosis, increasing the risk of death by two to three times after stroke. In this cohort, having AF and stroke recurrence slightly modified mortality risk^(24)^. It is also known that the prevalence of AF gradually increases with age and is a major cause of stroke in the elderly, being associated with high mortality rates^(29)^. In addition, AF is related to more severe strokes compared to other causes^(30)^.

The model stratified by NIHSS ≤ 13, reflecting the less severe cases, reinforced AF as a predictor and also showed a higher prior Rankin as a predictor, a variable that corroborates a Spanish study that identified that having a Rankin > 2 before stroke was a significant predictor of mortality for men aged 60 to 79 years^(5)^.

Since the severity of the event, added to risk factors such as age and diagnosis of AF, were shown to be the main predictors of death after a stroke, it should be noted that not only is the early search for a reference health service important for the reduction of mortality due to the event, but the primary prevention of stroke is required. Primary prevention strategies include community-based education programs, polypill, atrial fibrillation prevention and management, and digital health technology^(8)^.

The high number of people dying from stroke in Latin American countries requires concerted action by governments, health professionals, and other key concerned parties in the region to improve their primary prevention, acute care, and rehabilitation^(9)^.

## Study Limitations and Strengths

The limitations of this study include data collection performed in a single hospital in the state of Bahia, from the public network and reference for the care of people with ischemic stroke, which may keep specific characteristics of the sample; the identification of the extra-hospital death after 90 days being through telephone, resulting in follow-up losses; the reduced number of the sample and of participants in the stratified model with NIHSS ≥ 14, which may have compromised the results obtained in the associations of interest.

The main contribution of this study is to be the first to prospectively investigate the association of TARH with mortality in people with ischemic stroke and the relationship with variables of interest.

## CONCLUSIONS

The relationship between TARH and mortality up to 90 days after stroke was modified by the NIHSS. In the multivariate model stratified by NIHSS ≥ 14, higher TARH was associated with lower mortality, whereas in the model stratified by NIHSS ≤ 13, higher TARH was associated with higher mortality, but without statistical significance.

Having an AF diagnosis was a predictor of mortality in all models. Being older was a predictor of mortality in the model stratified by NIHSS ≥ 14. In addition, having a pre-stroke Rankin ≥ 3 was a predictor of mortality in the model stratified by NIHSS ≤ 13.

The association of the severity of the event and the presence of AF with mortality reinforces the importance of primary stroke prevention through health education actions and public policies aimed at identifying and taking action to control risk factors for the disease, recognizing its signs and symptoms, and better structuring of health care networks.
